# A clinical audit of anatomical side marker use in a pediatric medical imaging department: A quantitative and qualitative investigation

**DOI:** 10.1371/journal.pone.0242594

**Published:** 2020-11-24

**Authors:** Lilian Chung, Saravana Kumar, Joanne Oldfield, Maureen Phillips, Megan Stratfold

**Affiliations:** 1 UniSA Allied Health & Human Performance, University of South Australia, Adelaide, South Australia, Australia; 2 SA Medical Imaging, Women’s and Children’s Hospital, Adelaide, South Australia, Australia; University of Adelaide, AUSTRALIA

## Abstract

**Background:**

The presence of a radiopaque or digital anatomical side marker (ASM) is an important diagnostic feature on radiographs and should be a routine feature on every radiographic image. Despite its importance, research has indicated numerous instances where ASMs were absent which have the potential to lead to adverse events. To date, few studies have systematically examined the use of ASMs in clinical practice and explored medical imaging professionals’ perspectives on ASM use. This research aimed to address this knowledge gap.

**Methods:**

This investigation was conducted in two stages. Stage 1 involved a retrospective clinical audit of 421 randomly selected radiographs within 12-months at a pediatric medical imaging department. The data were analyzed for overall presence and type of marker use. Stage 2 comprised of semi-structured interviews with 11 radiographers to garner their perspectives on ASM use, and barriers and enablers to their use in clinical practice. The interviews were transcribed verbatim and thematically analyzed.

**Results:**

The overall presence of ASMs (radiopaque and digital) was observed on 99 per cent of radiographs. There was a noticeable shift towards the use of digital (78.8 per cent) compared to radiopaque ASMs (20.2 per cent), highlighting the growing trend towards using ASM in post-processing. A handful of images (*N* = 4) did not include any ASMs. Semi-structured interviews revealed multifaceted barriers (time, infection precautions, and patient factors) and few enablers (professionalism, legal requirement) for ASM use.

**Conclusion:**

This investigation, informed by quantitative and qualitative research paradigms, has shed new light on an important area of radiography practice. While missing ASMs were a small feature, there continue to remain opportunities where best practice standards can be improved. The increasing use of digital ASMs potentially highlights a shift in clinical practice standards.

## Introduction

Providing safe, quality health care is an important goal for any health service [1] and medical imaging is no exception. Within health care, general radiography has a versatile role in diagnosing various abnormalities, staging of diseases, and monitoring treatment outcomes [2]. As technology in medical imaging advances and general radiography continues to evolve in the digital world [3], it is vital that medical imaging professionals such as radiographers ensure their practice continues to be underpinned by quality standards. One of many components to consider when assessing a radiographic image for quality is the presence of an accurate anatomical side marker (ASM), which should be routinely labelled on every image [4].

An anatomical side marker (ASM) is a left (L) or right (R) marker which clearly indicates which side of the body is demonstrated on a radiograph [5]. In the past, these images were acquired using film-screen where the film had to be developed. To include an ASM using this system, a radiopaque marker needed to be contained in the primary field before exposure, or an anatomical side sticker was added onto the film after. With the introduction of digital radiology, radiographic images can be displayed on a screen electronically. There are currently two types of digital systems, computed radiography (CR) which involves using a cassette that is processed in an imaging plate reader before converting it to an electronic image, and digital radiography (DR) which omits this step and simultaneously displays the image on screen after exposure [6]. In current practice, an ASM can be included using a radiopaque ASM placed in the primary radiation field pre-exposure or digitally added post-exposure.

Between the two methods, using a radiopaque ASM pre-exposure is considered to be best practice [7, 8] as medico-legally, plain radiographs form part of a patient’s clinical record and are considered as legal documents [9]. As such, they require a radiopaque ASM to be within the primary radiation field for appropriate validation [10]. Radiographers are accountable for their actions [11] so best practice standards should be applied to every radiographic image given the possibility of them being used as evidence in a court of law [12]. Not only does this protect the radiographer, it also protects all stakeholders [10].

Despite the recognition for and the importance of having an ASM on radiographic images, an incorrect or missing ASM can be a common image labelling issue [13]. Errors can occur with both types of ASMs and potentially have adverse consequences such as misdiagnosis and wrong site surgery. Patient care could also be delayed if a repeat image is requested by the referring doctor due to error [5], consequently increasing the radiation dose to the patient [14].

Currently, only five studies have systematically quantified the use of ASMs in clinical practice [10, 11, 14–16]. These studies show that best practice standards are not always implemented in the clinical environment and that there are instances where ASMs are missing or incorrect. A seminal Australian study undertaken by Barry et al. [16] involved a retrospective clinical audit of 400 plain radiographs. This study found a small percentage of radiographic images (5.5 per cent) with missing ASMs where approximately half originated from images that were acquired using a portable machine out-of-department for patients aged 0–3 years. Although small, the potential for misadventure is still severe. Across the five studies, there were substantial variations in the percentage reported of missing ASMs on radiographs (1 per cent [10]—40.8 per cent [14]). This variation suggests complex factors likely influenced the use of ASMs on radiographs. Table 1 provides an overview of the current literature and its focus when investigating the use of ASMs on radiographs. Despite an emerging body of research on this topic, persistent knowledge gaps remain (Table 1). Informed by previous research by Barry et al [16], this research aimed to contribute to this evidence base by quantifying the use of ASMs in clinical practice and to explore radiographers’ perspectives regarding barriers and enablers to ASM use.

**Table 1:** Overview of the areas investigated for ASMs in current literature in chronological order.

	Finnbogason [15]	Aarke [13]	Platt [10]	Titley [14]	Adejoh [17]	Barry [16]	Attard [11]	This study
Presence of ASM	✓		✓	✓		✓	✓	✓
Frequency of the type of ASM used			✓	✓		✓	✓	✓
Placement of radiopaque ASM (radiation field)			✓	✓	✓			
Placement of radiopaque ASM (from guidelines)							✓	
Factors influencing ASM use			✓	✓		✓	✓	✓
Radiographer perspectives on ASMs							✓	✓
ASM related error rates		✓						
Interventions to improve ASM use		✓						

**Key:** ✓ = investigated by study.

**Abbreviation:** ASM, anatomical side marker.

## Methods

### Research question

This research aimed to address two key research questions: “How are anatomical side markers used in routine clinical practice?” and “What are radiographers’ perspectives on the barriers or enablers, if any exist, to influence the use of anatomical side markers?”

### Research design

This research used quantitative and qualitative research paradigms to acquire data in two separate, sequential stages to address each research question. The first stage involved a retrospective clinical audit of radiographic images at a pediatric medical imaging department which was conducted at the same institution as a previous study by Barry et al [16]. Following this, as part of second stage, a qualitative descriptive approach involving semi-structured interviews was undertaken at the same site to explore radiographers’ perspectives to the use of ASMs and the barriers and enablers to their use.

#### Stage 1: Quantitative

*Study design*. The first stage of the research involved a retrospective clinical audit of the use of ASMs over a 12-month period. While clinical audits cannot determine causality, they seek to improve the quality of health care and patient outcomes by evaluating data and measuring clinical performance against set criteria [18–20]. This process ensures health care services align with best practice and health care professionals adhere to the standards of their profession [19]. An audit tool designed by Barry et al [16] for their clinical audit at the same institution was used to inform the audit tool for this research.

*Inclusion/exclusion criteria*. Data were extracted from the institution’s radiology information system (RIS) used within the medical imaging department. The inclusion criteria consisted of in-department and out-of-department (portable) examinations for patients aged 0–18 years from July 2016 to June 2017. Excluded data included orthopantomograms (OPGs) and lateral cephalometric radiographs as the equipment used to obtain the images had an in-built ASM. Theatre cases and fluoroscopy studies were also excluded as it became apparent that radiopaque ASMs were not routinely used due to infection control risks and risks of obscuring anatomy during the procedure.

*Sample size*. Informed by previous calculation by Barry et al [16], a sample size of 400 radiographs was determined to be sufficient to attain relevant data (using a 5 per cent margin of error, a 95 per cent confidence interval and a population of 20,000 per annum). Therefore, this research too also used a similar sample size.

*Data collection*. In order to avoid selection bias, an independent information systems administrator extracted and de-identified the required data before it was accessed by the chief investigator on discs. As means of ensuring data capture over the 12-month period, a stratified systematic sampling approach was used per month [21, 22]. This process involved calculating how many radiographs were required to be sampled per month to attain the estimated sample size of 400 within the year (400 divided by 12 months) which equated to approximately 33 radiographs per month. Total number of radiographs for that month was divided by 33 to yield a monthly sampling interval. In order to obtain a sample of radiographs for that month, the radiographic examinations completed in that month was sorted in chronological order. After this, sampling was achieved by selecting every *n*^*th*^ examination. As one examination may contain more than one radiograph, we decided to include all radiographs within that selected examination to contribute to the total sample size. However, upon review of the extracted data, it was identified that the sample of radiographs obtained yielded a limited number of portable radiographic examinations. To ensure adequate coverage of both routine and portable radiographic examinations, re-sampling for portable radiographic examinations was undertaken. This was undertaken firstly by identifying the average percentage of portable radiographic examinations acquired per year. Using this percentage, the number of additional portable examinations required was determined (percentage of 400). A second sampling process using the same methodology was subsequently implemented to extract additional portable radiographic examinations.

*Data analysis*. This investigation utilized a clinical audit tool (S1 File) for the audit which was completed on site with the supervision of one of the members of the research team (who was also a member of staff at the institution). Microsoft Excel 2010 was used for data entry as well as for descriptive analyses. The data were analyzed for ASM presence and the type of ASM used. This was further analyzed for different parameters such as the projection type, body region, location the radiograph was acquired, and the system used to acquire the radiograph (CR or DR). These parameters were chosen based on previous research [14, 16] and in discussions with the research team comprising of radiographers with extensive clinical experience. The overall results were then categorized into three standards of practice: *best practice*, *acceptable practice and undesirable practice*. This investigation defined best practice standards as using a radiopaque ASM to annotate the correct side, acceptable practice as using a digital ASM to annotate the correct side or to correct a wrong or ambiguous radiopaque ASM, and undesirable practice as having no ASM present on the radiograph. These standards of practice were derived based on discussions with the research team comprising of radiographers with extensive clinical experience who were familiar with current practice standards.

*Reliability and validity*. Reliability was addressed by the adoption of an existing audit tool [17]. To further enhance accuracy, the audit tool was reviewed by a member of the research team who also provided feedback. This feedback was used to make minor modifications and enhance utility. In addition, a pilot audit of 46 de-identified radiographs (approximately 10 per cent of desired sample size) was completed three weeks prior to data collection to pilot test, validate the audit process, and to ensure all relevant data were appropriately captured. As part of this training process, during the interpretation stage, the chief investigator (who was a student radiographer at the time of this investigation) could seek clarification from a local expert (senior radiographer) on issues such as the type of ASM used, if it was placed in the correct position etc. This training was critical to ensure the chief investigator had adequate training and experience prior to undertaking the clinical audit.

#### Stage 2: Qualitative

*Study methodology*. While there are several qualitative methodologies, this research used a qualitative descriptive (QD) methodology. A QD approach is driven by straight descriptions utilizing the participant’s own language to obtain an accurate portrayal of people’s characteristics or circumstances [23, 24]. Given this research focused on radiographers’ perspectives of ASM use in clinical practice, this was the methodology of choice.

*Inclusion/exclusion criteria*. Eligible participants included qualified radiographers who had at least three months experience and mainly worked in the general radiology department which included portable examinations. Those who were not eligible were radiographers who exclusively worked with magnetic resonance imaging (MRI), computed tomography (CT) and/or angiography.

*Sample size*. Unlike quantitative research, sample size determination in qualitative research is not established using a sample size calculator [25]. Instead, it is based on ensuring adequate capture of the phenomena of interest balanced with time and resources available [24]. For the purpose of this research, all 25 eligible radiographers from the institution were invited to participate.

*Recruitment*. The total number of eligible radiographers within the department was relatively small (*N* = 25). Therefore, a sampling framework was not required as the sample was the same as the population of interest. Contact details of eligible participants were obtained through a member of the research team, who was also a staff member at the institution and the industry partner for this investigation. To avoid possible coercion, this team member was not involved in the recruitment process and merely forwarded the contact details of eligible participants to the chief investigator (as per agreed processes). The chief investigator, who was not employed by the institution and did not have any personal or professional relationship with the eligible participants, used the contact list to invite participants to the study (through email).

*Data collection*. Semi-structured interviews were used to garner the radiographers’ perspectives with open-ended questions focused on possible barriers, enablers and potential strategies to improve ASM use (S2 File). Semi-structured interviews allow for rich and meaningful exploration of a particular issue which was desired in this research [26]. To aid the transcription process, interviews were audio-taped after consent was obtained. The radiographers were de-identified during this process by allocating a random three-digit number acquired from a random number generator to each interview. Radiographers were also given the opportunity to withdraw from the study at any time.

*Data analysis*. The interviews were analyzed via thematic analysis, a systematic process to recognize patterns and identify themes [27]. Using the described steps by Green et al [28], the interviews were individually examined for relevant content that addressed the phenomena of interest. Codes were then applied to cluster information into thematic groupings. Patterns identified across the interviews were reassembled into overarching themes and further analyzed to address the research question [29].

*Trustworthiness and rigour*. As the qualitative research paradigm focusses on experiences and perspectives, it was important to ensure the data obtained was trustworthy. As means of ensuring trustworthiness and rigour, the following four components were addressed: how truthful the data were (credibility), how applicable the findings were (transferability), the degree the research could be repeated (dependability), and how close the data stayed true to the participant (confirmability) [25, 26]. Informed by Creswell and Miller [30], the following strategies were implemented. Credibility was addressed by the chief investigator repeating a summary of the participants’ responses for confirmation during the interviews. In addition, multiple coders were used during the data analysis stage. To address the transferability of results, rich descriptions were used to describe participants, the methods and how the data were collected. Prior to the interviews, reflectivity was also applied to refine questions and interview techniques through multiple pilot interviews for dependability and confirmability of results [31]. Confirmability was further addressed using direct quotes.

Ethical approval was sought and granted from the Women’s and Children’s Health Network Human Research Ethics Committee (HREC/17/WCHN/145) and the University of South Australia (ID: 200808) prior to the commencement of the study. Given the retrospective nature of the clinical audit, only written consent was obtained from all participants for the interview stage of the study.

## Results

### Stage 1: Quantitative

Four hundred and one randomly selected radiographs (168 examinations) were selected for audit over a 12-month period (July 2016 to June 2017). Due to a low number of portable radiographs present within the initial dataset, an additional 46 random portable radiographs were sampled, raising the total sample size to 447 randomly selected radiographs (206 examinations).

The research team excluded 26 radiographic images from the original sample, resulting in a final sample of 421. Reasons for exclusion were images which had missing data (*N* = 1), and individual segments of full spine or long leg examinations prior to electronic stitching to produce a full image (*N* = 25). Local protocol dictated that an ASM was only required on one region of the final stitched image and was not necessary for each individual segment. The inclusion of these radiographs would skew the dataset by erroneously inflating the number of radiographs with a missing ASM.

Agreed best practice standards for labelling the anatomical side of a radiographic image with a radiopaque marker (pre-exposure) was seen on 20.4 per cent of the radiographs audited. A digital ASM was present on 76.7 per cent of images and a small percentage (1.9 per cent) was annotated with both types of ASMs. Overall, ASMs were present on 99 per cent of radiographs. A small number of radiographs (*N* = 4) had missing ASMs and four had an ASM located on the incorrect side. Out of the four radiographs with missing ASMs, which came from different examinations, three were acquired in-department and one was acquired portably. The first radiograph did not have an ASM, however, a second image from the same examination had a digital ASM present. The second radiograph had used an incorrect radiopaque ASM, while all other images within the examination included a correct radiopaque ASM. The third and fourth radiographs had no ASM present and these were the only radiographs in this examination.

Table 2 highlights the type of ASM used overall and Table 3 demonstrates the percentage of adherence to practice standards in three areas. From Table 3, best practice standards were met 20.2 per cent of the overall radiographs and 23.5 per cent of radiographs obtained in-department. No radiographs acquired portably met best practice standards but did meet acceptable standards. Undesirable practice was observed in 0.8 per cent (3/365) of in-department radiographs and 1.8 per cent (1/56) for portable radiographs.

**Table 2:** Type of ASM used overall (*N* = 421).

	Radiopaque	Digital	Both	Incorrect/ absent	Marked appropriately
**Number of images**	86	323	8	8	413
**Frequency (%)**	20.4	76.7	1.9	1.9	98.1

**Abbreviation:** ASM, anatomical side marker.

**Table 3:** Standard of ASM use.

Practice Standard	Distribution of use (%)		
	Overall	In-department	Portable
Best	20.2	23.5	0
Acceptable	78.8	75.7	98.2
Undesirable	1.0	0.8	1.8
**Sample (*N*)**	421	365	56

**Abbreviation:** ASM, anatomical side marker.

**Fig 1:**
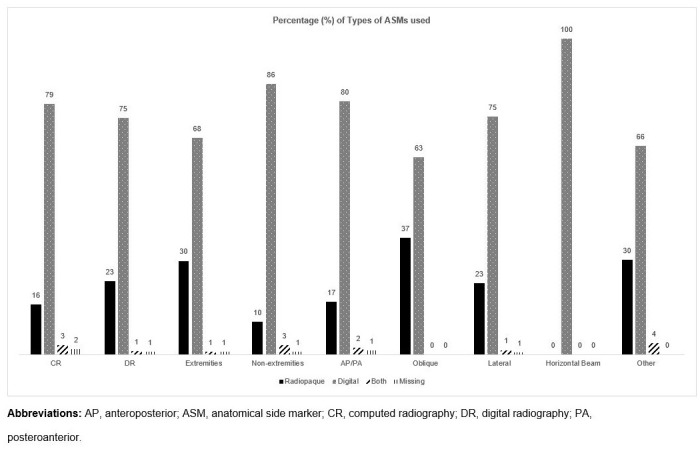
Percentage of the types of ASMs used for different parameters: Systems of radiograph acquisition (CR (*n* = 164) and DR (*n* = 257), body regions (extremities (*n* = 219) and non-extremities (*n* = 202)), and projection types (AP (anteroposterior)/PA (posteroanterior) (*n* = 216), oblique (*n* = 32), lateral (*n* = 137), horizontal beam (*n* = 9), and other views (swimmers, radial head, scaphoid, decubitus) (*n* = 27)).

Fig 1 demonstrates the frequency of the type of ASMs used for various parameters. For the acquisition systems, both computed radiography (CR) and digital radiography (DR) were comparable in terms of the type of ASM used. The results displayed that radiopaque ASMs were used approximately three times more in extremities compared to non-extremities and only digital ASMs were used for horizontal beam projections. For oblique projections, it was noted that the percentage of radiopaque ASM used was higher in comparison to the other projections. No ASMs were missing for oblique and horizontal bream projection.

### Stage 2: Qualitative

Out of the 25 eligible participants invited, 11 consented to participate in the interviews (n = 11). These participants had varying ranges of experience and areas of expertise (Table 4). All interviewed participants agreed that every radiograph should have some form of ASM. There was a mix of preferences for using radiopaque and digital ASMs where eight participants preferred to use radiopaque makers, two preferred to use digital, and one did not have a preference. While exploring radiographers’ perspectives regarding barriers and enablers of ASM use, several influencing factors were identified. There were often striking similarities in the factors featured for the barriers and enablers, possibly highlighting they were essentially “two sides of the same coin”. The following section summarizes these results.

**Table 4:** Participant characteristics.

Characteristics		Number
Experience	Less than 1 year	3
	1–5 years	3
	5–10 years	0
	Greater than 10 years	5
Professional experience	Pediatrics only	7
	Pediatrics/Adults	4
Area of expertise	General radiography only	5
	General radiography and another modality	6

#### Barriers to radiopaque ASM use

The radiographers highlighted numerous barriers to the use of ASMs in clinical practice and, radiopaque ASMs. These factors could be summarized into three themes: the *patient’s presentation* (the characteristics of the patient and how it influenced ASM use), *environmental or systemic factors* (environment or the working context surrounding the radiographer (e.g. department protocols, the technology used, and working conditions)), and the *radiographer’s attitude* (radiographer’s personal values, beliefs and habits).

*Patient presentation*. Most participants mentioned that the small size of the patient, especially in areas such as the neonatal intensive care unit (NICU) or the special care baby unit (SCBU) were barriers to the use of radiopaque ASMs. A senior radiographer (Participant 784) stated that:

“*I must admit, if I go to NICU or SCBU I don’t [use a radiopaque ASM], mainly because I think sometimes you’re doing a little 500g baby and you’re collimating down to that. Sometimes to actually get your marker on is a little bit… because they’re so small that I will admit, I don’t use them [radiopaque ASM] there.*”

Furthermore, chest radiographs were commonly requested at the NICU or the SCBU and another senior radiographer (Participant 745) mentioned risks of the radiopaque ASM covering anatomy due to patient movement or patient size. They elaborated by saying:

“*Sometimes the marker, if the patient moves, causes more problems because it ends up over the area you want to image and you end up doing a repeat, and that is probably the big reason why we don’t use [radiopaque] markers at all when we do portable chests… for neonates, the marker is almost sometimes as big as the child… the chances of you covering the marker with the baby is quite high.*”

Multiple participants highlighted other factors such as patient distress, time sensitive conditions, cognitive challenges, medical attachments, or infection control pre-cautions to influence their decision not to use radiopaque ASMs. These were mentioned by radiographers across all levels of experience. Examples of these situations are highlighted by three radiographers below:

“*…in the paediatric world, heaps of the cases you see are time sensitive, you got a kicking, screaming, crying baby that you’re trying to get a picture on and the last thing you worry about is [radiopaque] markers. There’s some instances where it’s just too hard to do.*”

–*Participant 184*

“*I don’t use them [radiopaque markers] if I’ve got a patient with pre-cautions… if the patient is getting distressed and it needs to be done quickly, I tend not to use them.*”

–*Participant 007*

“*Some children… [with] intellectual disability[ies]… they sometimes get distracted by you getting the [radiopaque] marker out or a little bit scared by you sticking [them] on the board so just to avoid that… if I think they’re going to be apprehensive, they’re already a bit stand offish, I just wouldn’t bother getting the marker out. It just kind of scares them because mine are actually coloured as well, that is a little bit more distracting too.*”

–*Participant 991*

*Environmental or systemic factors*. Factors such as the location of the examination, shift work, examination priorities (eg immobilization and infection control), and the accessibility of radiopaque ASMs were commonly mentioned as factors which precluded use. Most radiographers employed at the institution worked within a shift roster and one radiographer (Participant 184) mentioned:

“*…you don’t remember to put a marker on at 3 o’clock in the morning as you do at 3 o’clock in the afternoon.*”

Within NICU, examination priorities such as keeping the neonate still and maintaining controlled conditions seemed to be of a higher importance than the inclusion of a radiopaque ASM. The following comment supports this:

“*Getting the best quality image may not always include putting a [radiopaque] marker on, those examples where in NICU where you’re holding, you put your hands in the humidity crib… and you’re doing three things and then… adding a marker onto there can just mean: A, you take longer to do the x-ray, the crib’s open for longer… I just think in those instances, you get a better-quality image by putting them [digital ASMs] on afterwards.*”

–*Participant 499*

Time pressure environments such as the resuscitation department (resus) where radiographers may be subjected to a high-level trauma may also influence the use of radiopaque ASMs. One radiographer (Participant 634) expressed:

“*If you’re in a high-pressure trauma situation… I guess the need for precision and speed in your imaging and the intensity of the situation might preclude you from taking that step [to put on a radiopaque marker].*”

*Radiographer attitudes*. One barrier to the use of radiopaque ASMs stemmed from having the confidence and experience that pre-set imaging protocols were reliable and aligned with routine protocols for positioning. This was outlined for portable radiographs in NICU as an example because *“it [was] out of the ordinary doing something that [was] not AP flat [supine] here… mobiles [portables were] very much like that here [pediatric medical imaging department]”–Participant 499*.

The same participant further elaborates this point in a resuscitation environment and mentions that:

“*In a resus situation… we all know that the machine down there doesn’t flip the images, all the protocols are set to an AP [anteroposterior, patient facing x-ray beam] chest and you have to physically select a PA [posteroanterior, patient with back facing x-ray beam] chest from another menu which very rarely gets done in ED [emergency department]… I don’t think I’ve ever done a PA chest in resus, so you know you’re always doing an AP, you know it’s always going to display as an AP.*”

#### Enablers to radiopaque ASM use

The radiographers highlighted facilitators to the use of ASM in clinical practice and, radiopaque ASMs. These factors could be summarized into four (similar) themes: *patient’s presentation*, *environmental or systemic factors*, *radiographer attitudes* and *image related factors*. Image related factors referred to the resultant appearance and quality of the radiographic image.

*Patient presentation*. Most radiographers agreed that they would be more inclined to use a radiopaque ASM for patients who were co-operative, had no previous radiographs done, and could stay still. Co-operation was also deemed to be more likely for older children such as teenagers. This is highlighted by a radiographer (Participant 745) saying:

“*if they’re co-operative and [can] stay still then that’s great, you can put a [radiopaque] marker.*”

*Environmental or systemic factors*. Radiographers who preferred to use radiopaque ASMs reasoned that this preference was driven by an uncertainty or distrust in technology, medico-legal reasons, type of examination required, improved efficiency, and close access to the radiopaque ASMs. An example outlined by a recently qualified radiographer (Participant 007) expressed their sense of doubt towards technology and the patient’s anatomy in relation to their orientation by commenting:

“*I always prefer to use lead [radiopaque ASMs] just because it helps me to work out whether an image has been flipped… I never really know whether to trust the computer or trust [the] patient’s anatomy, so it’s easier to use lead.*”

This is also supported by a senior radiographer (Participant 745) outlining a similar ordeal:

“*… any work [done] PA [posteroanterior], especially chests, I always like to have a [radiopaque] marker on, or sinuses or skulls because if you do get dextrocardia or something like that… you can stand in front of the… film [detector] where your marker was and check, was it a left or right and then double check yourself that you have actually labelled it correctly. It’s too easy… just to sort of flip it [the x-ray image digitally] and go, that must be left…*”

Medico-legal reasons such as non-accidental injury (NAI) or mortuary cases were other factors which influenced the use of radiopaque ASMs. Having this knowledge seemed to increase the radiographers’ tendency to use radiopaque ASMs. One radiographer (Participant 310) said:

“*The reasons for using lead [radiopaque] markers for NAI or mortuary is [that] I know that they will go to court, so I always use them for that reason.*”

Some radiographers also commented that the use of radiopaque ASMs helped to speed up the examination and reduce chances of error by double checking their work. For instance, when queried why they used radiopaque ASMs, Participant 260 responded:

“*…it’s also like, a way of double checking things, like is this the left side? You double check the left side and then you put the left [radiopaque] marker on. Whereas, when on a computer and you [are] post-process[ing], chances are that… you rely on your memory and what’s on the request form so if something is wrong on the request form… or you did something wrong, there’s an increase chance of mistakes if you post-process that way.*”

As for participants who mainly used digital ASMs, they mentioned they would use radiopaque ASMs for bilateral examinations to avoid confusion during post-processing.

*Radiographer attitudes*. This was another interesting finding which consisted of a compilation of factors such as confidence, pride in work, developed habits, and showing a good standard. One radiographer (Participant 634) commented:

“*I guess it’s just taking pride in your work and having things labelled properly… I think it’s much more professional… it shows a good standard of practice I think, but I guess it’s just best practice. I pride myself taking the best images [I] can in each situation no matter how tricky it is so I think it’s always better to have it [radiopaque ASM] on there.*”

*Image related factors*. Other factors which enabled the use of a radiopaque ASM was that it was more permanent, and its presence was aesthetically pleasing. This was supported by a radiographer who had experience with both adult and pediatric patients (Participant 991):

“*I think aesthetically it looks nicer and I like that, well I know that someone can crop it off the [radiographic] image but I feel like [the radiopaque ASM] is more permanent.*”

## Discussion

To date, despite the potential for adverse consequences of missing or incorrect use of ASMs in clinical practice, research in this field continues to be in its infancy. Studies which have been conducted in this field are limited to a handful of quantitative studies with no research exploring radiographers’ perspectives of ASM use in clinical practice. This research used quantitative and qualitative research paradigms to examine the use of ASM in clinical practice (the “how many”?) and subsequently explored radiographers’ perspectives of ASM use, including barriers and enablers for its use (“the why”?). By doing so, this research has contributed to new knowledge in this field. The findings from this research highlight that in an overwhelming majority, radiographs did contain some type of ASM. This important positive finding is complemented by an interesting trend with the rise of digital ASMs. The preference for digital ASMs over radiopaque ASMs signifies a clear shift from acknowledged best practice. This research also sheds new light and provides some explanation for the barriers to the use of radiopaque ASMs. This may explain the increasing move towards digital ASMs.

This research identified only 1.9 per cent of radiographs with ASM errors (incorrect and missing). Although this only equates to eight out of 421 radiographs, these errors have the potential to result in serious and adverse outcomes for the patient. Previous research investigating the presence of ASMs have also identified similar results [10, 11, 16]. A unique characteristic of this research was that it was undertaken in a pediatric setting where the use of ASMs gains increasing significance due to the likelihood that these radiographs are the patient or child’s first. This highlighted an opportunity for improvement to ensure all radiographic images include an ASM.

Comparing the results of this research to Barry et al’s [16], where this investigation was also conducted and was a follow-on audit from Barry and collegues, there were fewer radiographs with missing ASMs (four vs 22). Additionally, this study found only one portable radiograph without an ASM, whereas Barry et al [16] found 12. Although this decrease is a positive finding, caution should be taken when making direct comparisons between the two studies due to the nature of both research designs (descriptive research with no ability to establish causality). A range of barriers for not using radiopaque ASMs were explored in the qualitative stage. Infection control was identified as one such a barrier to the use of radiopaque ASMs for portable radiographs and may explain the missing ASM in this case. ASMs were considered to transmit infections to potentially immunosuppressed patients. This reasoning is supported by Tugwell and Maddison [32] who found that radiopaque ASMs were potential carriers of micro-organisms. Alternatively, other factors which may have prevented the use of a radiopaque ASM could be examination priorities such as immobilization. Although radiographers have the option to include a digital ASM post examination, qualitative findings suggest that busy workloads and time pressures may play an influencing role here. These factors may have contributed to a radiograph with a missing digital ASM, resulting in an image without any side annotations. Nevertheless, missing ASM is a cause for concern, as not providing an ASM is undesirable practice, and should be avoided.

During the interviews, most of the radiographers agreed that the use of radiopaque ASMs was best practice, but the clinical audit found that there was a tendency to use digital ASMs compared to radiopaque for most investigated parameters. Despite knowing what best practice is, adherence was patchy. This finding was supported by several studies which described similar outcomes [10, 11, 16]. However, the findings of our research differed to a study by Titley and Cosson [14] who found that the use of radiopaque ASMs (34.5 per cent) was higher than digital (24.3 per cent). Without local insight into specific department expectations, it is difficult to explain this difference. It could be argued that the higher use of radiopaque ASMs within the primary beam in Titely’s and Cosson’s [14] study may be the result of more stringent managerial control and department auditing processes.

The preference to use digital ASMs perhaps highlights the increasing use of technology in health care and the convenience and the benefits it brings. While this might be the case, given that there continues to be opportunities for errors, there is an opportunity for radiography as a profession to come to a consensus on what is best practice in the use of ASMs. Without this, there will be a dichotomy on best practice standards (radiopaque ASM) and what occurs in the clinical environment (digital ASM).

An interesting finding from our study was that the percentage of radiopaque ASM use was higher in oblique views compared to the other projections. While this chance finding may have been due to random error, it is also likely due to a clinical reason. All oblique views were part of extremity examinations and some radiographers expressed a tendency to use radiopaque ASMs more frequently for these examinations. This may explain why the percentage of radiopaque ASMs observed for this projection is marginally higher in our study.

### Limitations

As with any research, this research also has some limitations. First, the data that formed part of the clinical audit was sourced from a system where it only included radiographs that had been archived for radiologist reporting. A possible scenario where a radiopaque marker was used but positioned outside the field of view was not captured by our study. Future studies which collect data prospectively could potentially provide opportunities to capture this data. Additionally, any radiographs rejected at the point of acquisition for ASM related errors (eg ASMs obscuring essential anatomy) were not captured in this sample and it may be possible that the reported errors in this research were underestimated. To address this issue, future research investigating a reject analysis on ASM related errors can be considered. Second, as part of this research also used qualitative research methodology and methods, it was important that strategies were implemented to ensure rigour and trustworthiness. These strategies include the use of direct quotes and reflectivity. Finally, the transferability of the qualitative data may also be limited as this research only included participants from a single site and within a specialized area (pediatrics). However, the rich description of participants, use of quotes and corresponding similar findings from other settings (as identified from other research) may assist in transferability.

## Conclusion

### Implications for practice and future research

Despite only a handful of radiographs with missing ASMs, this research highlighted the complexities which underpin the use of ASMs in clinical practice. While the use of radiopaque ASMs is best practice, the findings of this study, the first of its kind in this field, have identified several factors that have a positive and negative influence on their use. With the increased tendency to use digital ASMs, professional practice standards do not appear to have kept up with technology. Future research could explore what radiographers consider to be best practice, given the rise in popularity of digital ASMs and what role, if any, do radiopaque ASMs have in contemporary radiography.

## Supporting information

S1 File Clinical audit tool. (DOCX)

S2 File Question prompts. (DOCX)

## Abbreviations

AP: anteroposterior
ASM: anatomical side marker
CR: computed radiography
CT: computed tomography
DR: (direct) digital radiography
ED: emergency department
HREC: human research ethics committee
IT: information technology
MRI: magnetic resonance imaging
NAI: non-accidental injury
NICU: neonatal intensive care unit
OPG: orthopantomogram
PA: posteroanterior
PACS: picture archiving communication system
PRIMSA: Preferred Reporting Items for a Systematic Review and Metanalysis
QD: qualitative descriptive
RIS: radiology information system
SA: South Australia
SCBU: specialized care baby unit
UniSA: University of South Australia
WCHN: Women’s and Children’s Health Network
XR: x-ray
