# Supplementary material for: A clinical audit of anatomical side marker use in a pediatric medical imaging department: A quantitative and qualitative investigation
Source: PLoS One. 2020 Nov 24;15(11):e0242594. doi: 10.1371/journal.pone.0242594 (PMC7685512; doi:10.1371/journal.pone.0242594)
Supplement: S1 File — (DOCX) [file pone.0242594.s001.docx]

**S1 File. Clinical audit tool.**

An extract of the clinical audit tool used for data collection. Shows example of how the tool was filled for the following headings:

- Exam No.
- Image No.
- Anatomical Region
- Orientation
- Mode of x-ray
- CR/DR
- Marker present
- Radiopauqe marker visualised
- Digital marker visualised
- Radiopaque & digital marker visualised
- Is marker(s) correct
- Comments

**S1 Table. Extract of clinical audit tool.**

| **Exam. No.** | **Image No.** | **Anatomical Region** | **Orientation** | **Mode of x-ray** | **CR/DR** | **Marker present** | **Radiopaque marker visualized** | **Digital marker visualized** | **Radiopaque & digital marker present** | **Is marker(s) correct** | **Comments** |
| --- | --- | --- | --- | --- | --- | --- | --- | --- | --- | --- | --- |
| 3 | 1 | CHEST | AP SITTING | PORTABLE | CR | YES | NO | YES | NO | YES |  |
| 4 | 1 | CHEST | AP SUPINE | PORTABLE | CR | YES | NO | YES | NO | YES |  |
| 23 | 1 | CHEST | AP/PA | N/A | DR | YES | YES | NO | NO | YES |  |

**Abbreviations:** AP, anteroposterior; CR, computed radiography; DR, digital radiography; N/A, not applicable; PA, posteroanterior.
