# Supplementary material for: A clinical audit of anatomical side marker use in a pediatric medical imaging department: A quantitative and qualitative investigation
Source: PLoS One. 2020 Nov 24;15(11):e0242594. doi: 10.1371/journal.pone.0242594 (PMC7685512; doi:10.1371/journal.pone.0242594)
Supplement: S2 File — (DOCX) [file pone.0242594.s002.docx]

**S2 File. Question Prompts**

List of questions used to prompt the semi-structured interviews of the study. Questions asked covered the participant’s demographic, their perspective of using anatomical side markers, experience using anatomical side markers, and strategies to improve use of anatomical side markers.

1. **Participant demographic**
   - Can you tell me a little about yourself?
   - Can you tell me your qualifications and your professional background?
   - Tell me a little more about your working history (in medical imaging)?
2. **Perspectives of using anatomical side markers**
   - From your perspective, what are your views on anatomical side markers?
     1. Can you tell me about any instances where it was appropriate not to use markers in practice?
3. **Experience of using anatomical side markers**
   - What factors influence your decision to use anatomical side markers?
4. From your point of view, what barriers do you find when using anatomical side markers?
5. Can you tell me about any preferences you may have when using side markers?
6. What factors influence your choice of these preferences?
   - What are your thoughts on x-rays without anatomical side markers?
     1. What difficulties, if any, do you face when using radiopaque side markers?
7. **Strategies to improve use**
   - What suggestions would you make to improve the use of anatomical side markers?
     1. In your opinion, what kind of strategy to improve would be the most beneficial?
